# Completeness of reporting in abstracts of randomized controlled trials in dental medicine published from 2015–2023: A methodological study

**DOI:** 10.1371/journal.pone.0328271

**Published:** 2025-07-14

**Authors:** Nensi Bralić, Livia Puljak, Barbara Ćaćić, Israel Junior Borges do Nascimento, Marija Šimundić Munitić, Marina Krnić Martinić, Tonia Škoprc, Andrija Babić, Jelena Zekan, Ivana Vukičević, Tina Poklepović Peričić

**Affiliations:** 1 Department of Research in Biomedicine and Health, University of Split School of Medicine, Split, Croatia; 2 Center for Evidence-Based Medicine and Healthcare, Catholic University of Croatia, Zagreb, Croatia; 3 Faculty of Humanities and Social Sciences in Split, University of Split, Split, Croatia; 4 Division of Country Health Policies and Systems (CPS), World Health Organization Regional Office for Europe, Copenhagen, Denmark; 5 Zahnarztpraxis Tadic, Hülben, Germany; 6 Department of Otorhinolaryngology, University Hospital Center Split, Split, Croatia; 7 University of Split School of Medicine, Split, Croatia; 8 Institute of Emergency Medicine in Split-Dalmatia County, Split, Croatia; 9 Dental Center Dubravica, Vodice, Croatia; 10 University of Zagreb School of Dental Medicine, Zagreb, Croatia; 11 Department of Prosthodontics, Study of Dental Medicine, University of Split School of Medicine, Split, Croatia; Danube Private University, AUSTRIA

## Abstract

**Background:**

Well-reported scientific abstracts are essential as they provide a concise summary of key research findings. Our study aimed to assess the completeness of reporting in abstracts of randomized controlled trials (RCTs) in dental medicine using the CONSORT for Abstracts (CONSORT-A) checklist.

**Methods:**

This was a methodological cross-sectional study. We searched PubMed for RCTs published between August 2015 and August 2023 in Q1 journals from the “Dentistry, Oral Surgery & Medicine” category of the Journal Citation Reports. Two authors independently screened the records and assessed their adherence to 15 CONSORT-A items (items *Authors* and *Recruitment* were not assessed) for each abstract. All items were scored with ‘1’ if reported adequately (in line with CONSORT-A), ‘0’ if reported inadequately, and ‘0.5’ if the reporting was partially adequate. We calculated each abstract’s adherence (percentage) rate and median adherence across all abstracts. The adherence to CONSORT-A items was determined by dividing the number of articles that reported each item adequately by the total number of articles included. We also conducted a Jonckheere-Terpstra trend test to assess the temporal trend.

**Results:**

The search resulted in 1691 records, of which 1564 were eligible for inclusion. We analyzed a random sample of 400 abstracts. Median adherence to CONSORT-A overall was 43.3% (95% CI 43.3 to 43.3). Items with the highest adherence scores were ‘Conclusions’, ‘Objective’, and ‘Interventions’, while ‘Funding’, ‘Randomization’, and ‘Harms’ had the lowest adherence scores. Median adherence to CONSORT-A has not improved over time (p = 0.342). For each analyzed year, mean adherence to CONSORT-A was around 40%.

**Conclusions:**

Reporting in abstracts of RCTs in dental medicine was suboptimal, and there was no improvement from 2015 to 2023. Improved guideline enforcement and author education are vital for enhancing abstract reporting quality and transparency.

## Introduction

Randomized controlled trials (RCTs) support evidence-based practice by providing the highest level of evidence for making informed clinical decisions [1]. However, assessing the validity of RCTs is largely influenced by the quality of their reporting [2]. Previous studies found poor completeness of reporting of RCTs from different medical and dental medicine specialties [3–6]. Furthermore, due to journal article paywalls or lack of time, many clinicians do not read full texts when searching for evidence; instead, they use the information available in the abstracts [7]. However, relying only upon abstracts when making a clinical decision can become an issue, as the information in the abstracts is often limited or may not accurately display the information from the full text of the article [8–10]. This makes the reporting quality of abstracts of utmost importance.

The Consolidated Standards of Reporting Trials (CONSORT) reporting guidelines were developed to address the concerns regarding the quality of reporting in RCTs [11]. In 2008, a 17-item CONSORT for Abstracts (CONSORT-A) checklist was introduced as an extension to the CONSORT Statement [12]. The CONSORT-A checklist contains reporting recommendations for all abstract sections, including the aims, methods, results, and conclusions, to improve the reporting quality of journal and conference abstracts [12]. An acceptable threshold for adequate reporting, as shown by Plenković et al. [13], has commonly been set at ≥70% adherence in previous studies assessing reporting quality using standardized checklists, including CONSORT-based tools. However, there is limited evidence regarding how dental medicine abstracts of RCTs adhere to the CONSORT-A guidelines. Previous studies have underlined the importance of improving the quality of reporting for RCT abstracts from some specific dental medicine specialties, including periodontology, orthodontics, and pediatric dentistry [14–16]. However, there is a gap in the literature regarding the overall completeness of reporting in abstracts across all dental medicine specialties.

While evidence exists that reporting quality of RCT abstracts has been assessed and shown to be suboptimal in other fields of medicine, such as psychiatry, nursing, occupational therapy, and critical care [17–20], dental medicine could have particular characteristics that justify a separate evaluation. Dental clinical trials often involve smaller sample sizes, different intervention modalities (e.g., procedures on teeth, implants, or restorations rather than whole-body treatments), and distinctive trial designs like split-mouth designs, which may influence reporting practices. Furthermore, dental research communities might not have as strong a tradition of adherence to general reporting guidelines compared to some larger medical disciplines. Therefore, our study was necessary not simply to replicate previous findings from other fields, but to specifically assess whether abstracts in dental medicine meet expected reporting standards, and to identify areas for improvement tailored to the unique aspects of this discipline.

The null hypotheses of the study were:

H₀-1: The median adherence to the CONSORT-A checklist in randomized controlled trial abstracts in dental medicine is equal to or greater than an acceptable threshold of completeness of 70%.

H₀-2: There is no significant difference in median adherence to the CONSORT-A checklist over time.

## Materials and methods

### Study design

This was a cross-sectional, methodological, research-on-research study. The protocol for this study had been registered on the Open Science Framework on 22 April 2020 before study commencement (https://osf.io/kwn6v).

### Eligibility criteria

We included abstracts of articles reporting results of RCTs published in journals indexed in the subject category “Dentistry, Oral Surgery & Medicine” from the Journal Citation Reports (JCR) belonging to the Journal Impact Factor (JIF) quartile Q1 in August 2023. A list of 24 eligible journals is listed in S1 Appendix. Eligible designs of RCTs were parallel-group, cluster, cross-over, factorial, and split-mouth designs. We excluded studies that did not mention randomization in any part of the article, including the title, abstract and the full text of the article. We also excluded studies on cost-effectiveness or diagnostic test accuracy, studies not conducted on humans, studies that did not have an abstract, in vitro studies, and methodological studies. We used the STROBE reporting checklist for reporting (S2 Table).

### Search

We searched PubMed using the advanced search feature. The search was restricted to RCTs by using the publication type filter and conducted on August 1st, 2023. To retrieve studies published during the targeted eight years, we used the date of publication filter from August 1st, 2015, to August 1st, 2023. This period was chosen because we wanted to analyze a more recent sample of abstracts. There were no restrictions regarding the language of publication. A complete search strategy is provided in S3 Appendix.

### Screening

The Rayyan web application [21] was used for title, abstract, and full-text screening. Full texts were screened for studies whose abstracts did not provide enough information to determine whether or not a study was randomized. Each record was screened independently by two authors. Three members of the author team (NB, BĆ, TPP) participated in screening. Disagreements were resolved through discussion between the same three authors at the end of the screening process.

### Sample calculation

We used an online sample size calculator (https://www.calculator.net/sample-size-calculator.html) to determine the minimum number of abstracts required to accurately represent the problem and to allow reliable statistical analysis. By setting the confidence level to 95%, the margin of error to 5%, the population proportion to 50%, and the population size to 1564, we calculated that we would need a minimum of 309 abstracts.

We then used a random number generator (https://www.random.org/integer-sets) to select a random sample of 400 abstracts for this study. First, all eligible abstracts identified through the PubMed search were assigned a unique numerical identifier, ordered sequentially from 1 to the total number of eligible abstracts. Using the “Integer Set Generator” tool at random.org, we generated a random set of 400 unique integers, specifying the minimum value as 1 and the maximum value as the total number of eligible abstracts, without allowing duplicate numbers. The list of randomly selected integers corresponded to the abstracts included in the sample for detailed analysis. This procedure ensured that the sampling process was unbiased and reproducible.

### Data extraction

Data was extracted from each eligible abstract in duplicate and independently by pairs of authors (NB, BĆ, IJBN, MŠM, MKM, TŠ, AN, JZ, IV, TPP). We created a data extraction form in Excel (Microsoft, Redmond, WA, USA) specifically for this study. Data items extracted from each of the 400 abstracts are presented in Table 1.

**Table 1:** Data items extracted from each abstract.

Item		Description
Study ID		Number of the abstract (1–400)
Title		Title of the trial
Year		Year of publication
Journal		Name of the journal that published the trial
CONSORT - A	Title	Identification of the study as randomized
	Trial design	Description of the trial design (e.g., parallel, cluster)
	Methods	
	Participants	Eligibility criteria for participants and the settings
	Interventions	Interventions intended for both groups
	Objective	Specific objective(s) of the trial
	Outcome	Pre-specified assessment or measurement to address the objective(s)
	Randomization	How participants were allocated to interventions
	Blinding (masking)	Whether or not participants, caregivers, and those assessing the objective(s) were blinded to group assignment
	Results	
	Numbers randomized	Number of participants screened and randomized to each group
	Numbers analyzed	Number of participants analyzed in each group
	Outcome	Results for the objective(s); including expressions of uncertainty
	Harms	Important adverse events or side effects
	Conclusions	General interpretation of the results of the trial
	Trial registration	Registration number and name of the registry
	Funding	Source of funding for the trial
Field of dentistry		Specific dental medicine specialty (e.g., periodontology, endodontics)
Unit of randomization		What was randomized (e.g., participants, teeth, implants)
Design of RCT		Trial design (e.g., parallel, cluster, cross-over, split-mouth)

Data extractors used the CONSORT-A checklist guide as a reference [12]. CONSORT-A has 17 items, but for this study, we assessed 15 items. The item ‘Authors’ was not assessed as it was specific to conference abstracts. Also, the item ‘Recruitment’ was excluded as all included studies were completed, so the item did not apply to our sample. After data extraction, independently extracted data was compared, and discrepancies were resolved through discussion or by senior authors (TPP, LP).

### Assessment of reporting completeness

The completeness of reporting of included abstracts was evaluated by checking whether the criteria for 15 CONSORT-A items were met adequately. We scored an individual item as “1” if it was reported adequately, “0” if it was not reported at all, and “0.5” if the reporting was partially adequate. A description of what was missing was marked for partially reported items.

### Data analysis

Descriptive summary statistics were calculated for each categorical variable, reported as frequencies and percentages. A percentage adherence was calculated for each abstract by dividing the sum of scores from each item by the number of items (N = 15), and median adherence was calculated across all abstracts. The adherence to each CONSORT-A item was determined by dividing the number of articles that reported each item adequately (e.g., score “1”) by the total number of articles included. We used the Kolmogorov-Smirnov (KS) test to check the normality of data distribution for numerical variables. As the data was not normally distributed, the temporal trend was assessed using the Jonckheere Terpstra trend test, a test appropriate for assessing ordered trends across independent groups [22]. We used the Kruskal-Wallis test to assess the association between adherence to CONSORT-A and study design as well as the dental medicine specialty, followed by Dunn’s *post hoc* test to compare individual categories. Statistical significance was set at p < 0.05. We used MedCalc version 22.023 (MedCalc Software, Ostend, Belgium, https://www.medcalc.org) and JASP software, version 0.18.3 (JASP Team, 2022, https://jasp-stats.org) for statistical analyses.

### Differences between the protocol and results

The study was originally planned to include abstracts published from 2015 to 2020, as described in the preregistered protocol (https://osf.io/kwn6v). The study was resumed in August 2023 with an extended time frame (up to August 2023). The study design was not changed. The only change between the protocol and the final study was related to the timeframe when the analyzed trials were published.

## Results

**Fig 1:**
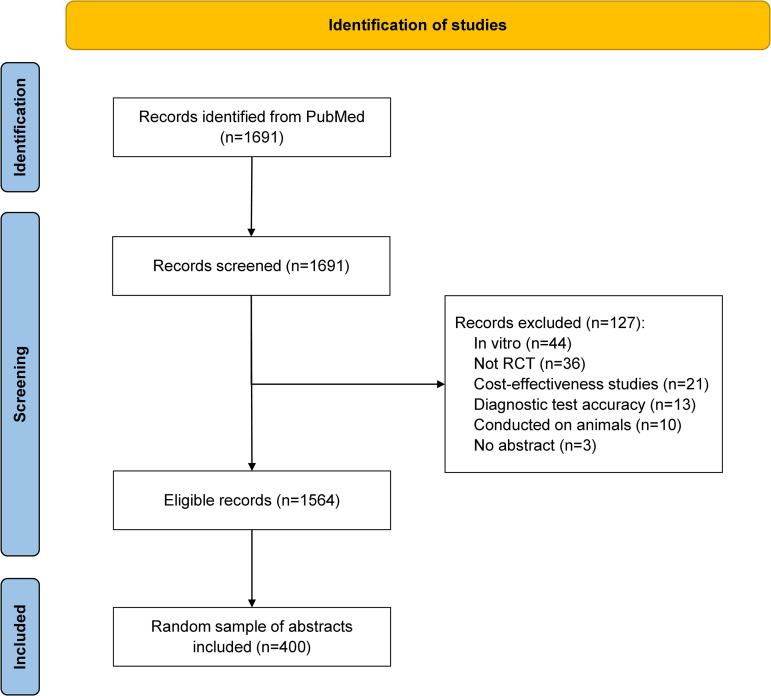
Abstract selection flowchart.

A total of 1,691 records were retrieved from PubMed. After the screening phase, a total of 127 publications were considered ineligible based on their titles, abstracts, and full texts. From the remaining 1,564 abstracts that met the criteria for inclusion, we selected a random sample of 400 abstracts for further analyses (Fig 1).

### Trial characteristics

Most trials in the sample were published in the *Journal of Clinical Periodontology*, *Clinical Oral Implants Research*, and the *Journal of Dentistry*. RCTs in our sample primarily employed a parallel-group design, with human participants being the units of randomization. The main fields of dental medicine were periodontology, oral surgery and implantology, and endodontics (Table 2).

**Table 2:** Characteristics of included trials.

Characteristics	Total (%), N = 400
**Journal**	
Journal of Clinical Periodontology	60 (15.00)
Clinical Oral Implants Research	49 (12.25)
Journal of Dentistry	48 (12.00)
Clinical Implant Dentistry and Related Research	47 (11.75)
Journal of Periodontology	47 (11.75)
Journal of Endodontics	26 (6.50)
Journal of Prosthetic Dentistry	17 (4.25)
Oral Diseases	17 (4.25)
International Journal of Paediatric Dentistry	15 (3.75)
International Endodontic Journal	14 (3.50)
Caries Research	9 (2.25)
Journal of Dental Research	8 (2.00)
Oral Oncology	8 (2.00)
Journal of Prosthodontic Research	7 (1.75)
Journal of Evidence-Based Dental Practice	6 (1.50)
European Journal of Paediatric Dentistry	5 (1.25)
Journal of Prosthodontics-Implant Esthetic and Reconstructive Dentistry	5 (1.25)
Progress in Orthodontics	5 (1.25)
Dental Materials	4 (1.00)
Journal of the American Dental Association	2 (0.50)
International Journal of Oral Science	1 (0.25)
Japanese Dental Science Review	0 (0.00)
Periodontology 2000	0 (0.00)
Seminar in Orthodontics	0 (0.00)
**Year of publication**	
2015	17 (4.25)
2016	47 (11.75)
2017	56 (14.00)
2018	51 (12.75)
2019	58 (14.50)
2020	54 (13.50)
2021	45 (11.25)
2022	46 (11.50)
2023	26 (6.50)
**Type of a randomized controlled trial**	
Parallel-group	323 (80.75)
Split-mouth	37 (9.25)
Cross-over	35 (8.75)
Cluster	4 (1.00)
Factorial	1 (0.25)
**Unit of randomization**	
Participants	299 (74.75)
Sites	40 (10.00)
Teeth	32 (8.00)
Implants	19 (4.75)
Tooth restorations	10 (2.50)
**Field of dentistry**	
Periodontology	115 (28.75)
Oral surgery and implantology	101 (25.25)
Endodontics	47 (11.75)
Prosthodontics	30 (7.50)
Pediatric dentistry	28 (7.00)
Oral medicine	26 (6.50)
Preventive dentistry	19 (4.75)
Restorative dentistry	18 (4.50)
Orthodontics	8 (2.00)
Aestetic dentistry	8 (2.00)

### Adherence to CONSORT-A

**Fig 2:**
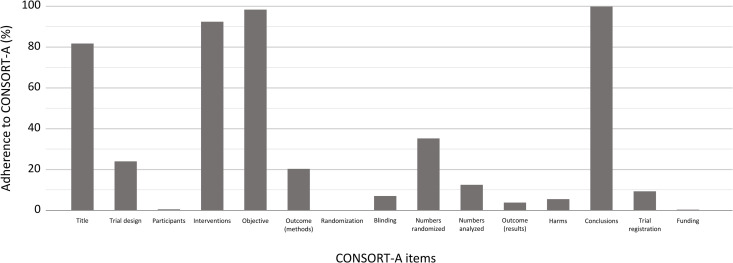
Overall adherence to the 15 analyzed CONSORT-A items.

Median adherence to CONSORT-A across all abstracts was 43.33% (95% CI 43.33 to 43.33). Adherence to each CONSORT-A item is presented in Table 3. The most adequately reported items were ‘Conclusions’, ‘Objective’, and ‘Interventions’, while ‘Funding’, ‘Randomization’, and ‘Harms’ were reported most poorly (Fig 2). There were no statistically significant differences in adherence to CONSORT-A between trials with different units of randomization (p = 0.910). However, there were differences in adherence between parallel-group design and cluster and split-mouth designs and between cluster and cross-over design, with parallel-group designs having lower adherence (43.33%, IQR 36.67 to 46.67) compared to cluster (55.00%, IQR 50.00 to 60.00) and split-mouth (46.67%, IQR 43.33 to 50.83) designs and cross-over design (43.33%, IQR 40.00 to 50.00) having lower adherence compared to cluster design.

**Table 3:** Adherence to 15 analyzed CONSORT-A items (N = 400).

Item	Adequately reported (score = 1), n (%)	Partially reported (score = 0.5), n (%)	Not reported (score = 0), n (%)
Title	327 (81.75)	0 (0.00)	73 (18.25)
Trial design	96 (24.00)	0 (0.00)	304 (76.00)
Methods			
Participants	2 (0.50)	298 (74.50)	100 (25.00)
Interventions	370 (92.50)	28 (7.00)	2 (0.50)
Objective	394 (98.50)	0 (0.00)	6 (1.50)
Outcome	81 (20.25)	318 (79.50)	1 (0.25)
Randomization	0 (0.00)	13 (3.25)	387 (96.75)
Blinding (masking)	28 (7.00)	81 (20.25)	291 (72.75)
Results			
Numbers randomized	141 (35.25)	230 (57.50)	29 (7.25)
Numbers analyzed	50 (12.50)	65 (16.25)	285 (71.25)
Outcome	15 (3.75)	219 (54.75)	166 (41.50)
Harms	22 (5.50)	1 (0.25)	377 (94.25)
Conclusions	400 (100.00)	0 (0.00)	0 (0.00)
Trial registration	37 (9.25)	16 (4.00)	347 (86.75)
Funding	1 (0.25)	0 (0.00)	399 (99.75)

### Temporal trend in adherence to CONSORT-A

**Fig 3:**
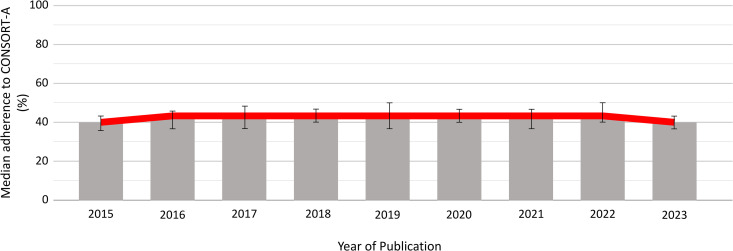
Adherence to CONSORT-A trend across years.

Median adherence to CONSORT-A among abstracts from dental medicine RCTs has not improved over time (p = 0.342). In each analyzed year, mean adherence to CONSORT-A was around 40% (Fig 3).

## Discussion

In this study, we hypothesized (H_0_-1) that the median adherence to the CONSORT-A checklist in abstracts of RCTs in dental medicine would be equal to or greater than an acceptable threshold of 70%. However, based on our findings, H_0_-1 was rejected, as the median adherence to CONSORT-A in our study was substantially below the 70% threshold. This indicates that, similarly to observations in other fields, reporting quality in abstracts of RCTs in dental medicine remains suboptimal.

Our study showed suboptimal completeness of reporting in abstracts of RCTs in the highest-ranking journals in dental medicine, with several CONSORT-A items, such as ‘Randomization’, ‘Harms’, and ‘Funding’ being reported exceptionally poorly. Additionally, authors often omitted crucial information like the location and time frame for the trial (required for item Participants of the CONSORT-A checklist), relevant outcome measures, methods used to randomize participants and conceal allocation, as well as the trial’s design, making it challenging for readers to assess whether the research aligns with what they were looking for. Several studies analyzed the reporting completeness of RCT abstracts from different medical fields and discovered comparable limitations of those abstracts [17,18,23]. We also observed differences in adherence to CONSORT-A between abstracts of RCTs among different trial designs. We found that abstracts of RCTs with parallel-group designs had significantly lower adherence to CONSORT-A than cluster or split-mouth designs, while cross-over designs showed lower adherence to CONSORT-A than cluster study designs. This may be attributed to the researchers’ greater familiarity with parallel-group and cross-over designs, resulting in a less rigorous approach to reporting.

Furthermore, we found that the reporting quality of dental medicine RCT abstracts has not improved from 2015 to 2023. Therefore, the second null hypothesis (H_0_-2) was not rejected. This is not in line with the findings for RCT abstracts in nursing, psychiatry, and critical care, which showed that the reporting quality has somewhat improved over time since the introduction of the CONSORT-A checklist in 2008 [18–20]. Adherence to relevant reporting guidelines is primarily expected from the authors as they are responsible for the quality of their work. However, the poor reporting quality of dental medicine RCT abstracts points to shortcomings in the peer review and publication process within the field. The responsibility for checking the reporting quality of the manuscript lies with the reviewers. Additionally, the Handling Editor is expected to verify compliance with all relevant reporting guidelines followed by the Editor-in-Chief, who gives the final approval.

Furthermore, different journals impose different word count limits for abstracts, with some allowing only up to 250 words, while others may be more flexible. It is more likely that more extended abstracts would be able to address the CONSORT-A criteria better. This variance in the allowed word count could contribute to the reporting quality of the abstracts, irrespective of the authors’ intentions to follow the guidelines.

The importance of consistency in standardized reporting of abstracts extends to the researchers producing systematic reviews who, during the screening process, often look at the abstracts to decide whether to include studies in their review. If the information in the abstract is lacking, a trial that should be included in the review could be discarded, affecting the overall review’s findings [24].

While our study provides a comprehensive assessment of the reporting quality of RCT abstracts from various dental medicine specialties, it is important to recognize its limitations. We included only trials published in Q1 journals. However, limiting ourselves to Q1 journals might have excluded high-quality RCTs published in journals in lower JCR quartiles. Furthermore, while two independent assessors scored adherence to each CONSORT-A item to increase accuracy and reduce potential bias, discrepancies in the subjective interpretations are always a possibility. Moreover, we did not account for potential differences in journal-specific guidelines and policies that could hinder adherence to CONSORT-A items. Future studies could expand the scope of journal inclusion and consider searching additional bibliographic databases such as Embase or Scopus to capture studies published in journals not indexed in PubMed. Additionally, a deeper qualitative investigation into the reasons for such poor reporting quality of dental medicine RCT abstracts should be conducted. Some explanatory variables that could be taken into consideration include journal endorsement of the CONSORT-A checklist and income level or region of the world where the study was conducted to understand better the impact of contextual differences on abstract reporting quality.

Our study also has several important strengths. Including a large number of abstracts from various dental medicine specialities allowed us to have a comprehensive overview of the reporting practices across the field. Another strength of our study is the extended time frame, allowing for the analysis of a trend in reporting quality over nearly a decade. Finally, to ensure the precision and objectivity of findings, every abstract was assessed by two independent authors in both screening phases and the data extraction phase.

## Conclusions

Our study showed that abstracts of RCTs published in the highest-ranking dental medicine journals adhered poorly to the CONSORT for Abstracts guidelines. There is a need to improve the reporting of several crucial aspects of abstracts in dental medicine publications, particularly regarding outcome measures, trial design, blinding, and randomization. It is also concerning that the reporting quality has not improved from 2015 to 2023, highlighting a growing need for interventions aimed at ensuring better adherence to the CONSORT-A checklist in dental medicine RCT abstracts. This could be achieved by stricter enforcement of CONSORT-A guidelines by the journals, providing training programs focused on teaching proper abstract reporting, instructing peer reviewers to assess adherence to the CONSORT-A checklist as part of the review process and allowing for a more flexible word count for abstracts to facilitate more transparent reporting of dental medicine RCT abstracts.

## Supporting information

S1 Appendix List of eligible journals. (DOC)

S2 Table STROBE checklist. (DOC)

S3 Appendix PubMed search strategy. (DOC)
