# Supplementary material for: Completeness of reporting in abstracts of randomized controlled trials in dental medicine published from 2015–2023: A methodological study
Source: PLoS One. 2025 Jul 14;20(7):e0328271. doi: 10.1371/journal.pone.0328271 (PMC12258550; doi:10.1371/journal.pone.0328271)
Supplement: S1 Appendix — (DOC) [file pone.0328271.s001.doc]

**S1 Appendix.** List of eligible journals

A list of eligible journals indexed in the subject category "Dentistry, Oral Surgery & Medicine" from the Journal Citation Reports (JCR) belonging to the Journal Impact Factor (JIF) quartile Q1 in August 2023.

1. Periodontology 2000
2. International Journal of Oral Science
3. Journal of Dental Research
4. Journal of Clinical Periodontology
5. Japanese Dental Science Review
6. International Endodontic Journal
7. Dental Materials
8. Progress in Orthodontics
9. Oral Oncology
10. Journal of Prosthetic Dentistry
11. Journal of Dentistry
12. Journal of Periodontology
13. Clinical Oral Implants Research
14. Journal of Endodontics
15. Seminars in Orthodontics
16. Caries Research
17. Journal of Prosthodontics-Implant Esthetic and Reconstructive Dentistry
18. Journal of the American Dental Association
19. Oral Diseases
20. International Journal of Paediatric Dentistry
21. Journal of Prosthodontic Research
22. Clinical Implant Dentistry and Related Research
23. European Journal of Paediatric Dentistry
24. Journal of Evidence-Based Dental Practice
