# Supplementary material for: Completeness of reporting in abstracts of randomized controlled trials in dental medicine published from 2015–2023: A methodological study
Source: PLoS One. 2025 Jul 14;20(7):e0328271. doi: 10.1371/journal.pone.0328271 (PMC12258550; doi:10.1371/journal.pone.0328271)
Supplement: S3 Appendix — (DOC) [file pone.0328271.s003.doc]

**S3 Appendix.** PubMed search strategy

PubMed search strategy undertaken August 1st, 2023.

((((((((((((((((((((((("Periodontology 2000"[Journal]) OR ("International journal of oral science"[Journal])) OR ("Journal of dental research"[Journal])) OR ("Journal of clinical periodontology"[Journal])) OR ("The Japanese dental science review"[Journal])) OR ("International endodontic journal"[Journal])) OR ("Dental materials : official publication of the Academy of Dental Materials"[Journal])) OR ("Progress in orthodontics"[Journal])) OR ("Oral oncology"[Journal])) OR ("The Journal of prosthetic dentistry"[Journal])) OR ("Journal of dentistry"[Journal])) OR ("Journal of periodontology"[Journal])) OR ("Clinical oral implants research"[Journal])) OR ("Journal of endodontics"[Journal])) OR ("Seminars in orthodontics"[Journal])) OR ("Caries research"[Journal])) OR ("Journal of prosthodontics : official journal of the American College of Prosthodontists"[Journal])) OR ("Journal of the American Dental Association (1939)"[Journal])) OR ("Oral diseases"[Journal])) OR ("International journal of paediatric dentistry"[Journal])) OR ("Journal of prosthodontic research"[Journal])) OR ("Clinical implant dentistry and related research"[Journal])) OR ("European journal of paediatric dentistry"[Journal])) OR ("The journal of evidence-based dental practice"[Journal]) AND ((Randomized Controlled Trial[ptyp]) AND (2015/8/1:2023/8/1[pdat]))
